# Key role of integrin α_IIb_β_3_ signaling to Syk kinase in tissue factor-induced thrombin generation

**DOI:** 10.1007/s00018-012-1033-2

**Published:** 2012-06-06

**Authors:** Paola E. J. van der Meijden, Marion A. H. Feijge, Frauke Swieringa, Karen Gilio, Reyhan Nergiz-Unal, Karly Hamulyák, Johan W. M. Heemskerk

**Affiliations:** 1 Department of Biochemistry, Cardiovascular Research Institute Maastricht (CARIM), Maastricht University, PO Box 616, 6200 MD Maastricht, The Netherlands; 2 Haemostasis Laboratory, Department of Internal Medicine, Maastricht University Medical Centre, Maastricht, The Netherlands

**Keywords:** Platelets, Integrin α_IIb_β_3_, Thrombin generation, Syk kinase, PS exposure

## Abstract

The fibrin(ogen) receptor, integrin α_IIb_β_3_, has a well-established role in platelet spreading, aggregation and clot retraction. How α_IIb_β_3_ contributes to platelet-dependent coagulation is less well resolved. Here, we demonstrate that the potent suppressing effect of clinically used α_IIb_β_3_ blockers on tissue factor-induced thrombin generation is linked to diminished platelet Ca^2+^ responses and phosphatidylserine (PS) exposure. The same blockers suppress these responses in platelets stimulated with collagen and thrombin receptor agonists, whereas added fibrinogen potentiates these responses. In platelets spreading on fibrinogen, outside-in α_IIb_β_3_ signaling similarly enhances thrombin-induced Ca^2+^ rises and PS exposure. These responses are reduced in α_IIb_β_3_-deficient platelets from patients with Glanzmann’s thrombasthenia. Furthermore, the contribution of α_IIb_β_3_ to tissue factor-induced platelet Ca^2+^ rises, PS exposure and thrombin generation in plasma are fully dependent on Syk kinase activity. Tyrosine phosphorylation analysis confirms a key role of Syk activation, which is largely but not exclusively dependent on α_IIb_β_3_ activation. It is concluded that the majority of tissue factor-induced procoagulant activity of platelets relies on Syk activation and ensuing Ca^2+^ signal generation, and furthermore that a considerable part of Syk activation relies on α_IIb_β_3_ signaling. These results hence point to a novel role of Syk in integrin-dependent thrombin generation.

**Electronic supplementary material:**

The online version of this article (doi:10.1007/s00018-012-1033-2) contains supplementary material, which is available to authorized users.

## Introduction

Integrin α_IIb_β_3_ (glycoprotein IIb/IIIa) is among the most abundantly expressed glycoproteins at the platelet surface, which strongly regulates the adhesion and aggregation of platelets. Platelet agonists induce a conformational change in the extracellular part of α_IIb_β_3_ via so-called inside-out signaling events, accumulating in Rap1b activation and talin complex formation, which increases the adhesiveness of the integrin [1–3]. Conversely, binding of α_IIb_β_3_ to its ligands, such as fibrinogen, fibrin and von Willebrand factor, stimulates outside-in signal transduction by a pathway involving activation of Src and Syk protein tyrosine kinases [4–6]. Platelet responses known to rely on α_IIb_β_3_ outside-in signaling include the shedding of microparticles, the formation of lamellipods of fibrinogen-adhered platelets, and the contraction of platelets within a fibrin clot [7–9].

It has been known for a long time that platelets stimulated with strong agonists promote the processes of thrombin generation and blood coagulation [10, 11]. This procoagulant function is triggered by agonist combinations like collagen/thrombin, which evoke prolonged and high rises in cytosolic [Ca^2+^]_i_, and also by stimulating platelets in plasma with tissue factor [12, 13]. The procoagulant activity is caused by exposure of the negatively charged phosphatidylserine (PS), at the membrane surface via a transmembrane protein encoded by TMEM16F [14], which promotes the local assembly of vitamin K-dependent coagulation factors and hence generation of thrombin [15]. Early findings have pointed to a significant role of α_IIb_β_3_ in the development of platelet procoagulant activity, in that integrin antagonists were found to suppress tissue factor-induced thrombin generation [12, 16, 17]. Yet, the mechanism by which α_IIb_β_3_ blockage interferes with platelet-dependent coagulation has not been resolved. Suggestions are that the integrin is (1) involved in the formation of procoagulant microparticles [17–19], (2) directly binds prothrombin [20], or (3) provides binding sites for factor Va and other coagulation factors [21, 22]. Studies so far are complicated by data that show that distinct α_IIb_β_3_ antagonists may differ in their effects on platelet activation and procoagulant activity [19, 23]. Another complicating factor is that part of the role of α_IIb_β_3_ may be secondary to that of autocrine ADP, which enhances PS exposure via P2Y_12_ receptor stimulation [24–26].

In this paper, we hypothesize that α_IIb_β_3_ interaction with its principal ligand, fibrinogen, enhances platelet-dependent thrombin generation via an outside-in signaling mechanism. We demonstrate that signaling via Syk kinase is responsible for the majority of tissue factor-induced thrombin generation of platelets in plasma by stimulating PS exposure.

## Materials and methods

### Materials

Human α-thrombin was obtained from Enzyme Research Laboratories, recombinant human tissue factor came from Dade Behring, abciximab (reopro) from Centocor; tirofiban (aggrastat) from Merck Sharp & Dohme, and eptifibatide (integrilin) from GlaxoSmithKline. Dimethyl BAPTA (DM-BAPTA), Fura-2 and Fura-Red acetoxymethyl esters were from Molecular Probes, while Syk inhibitor II and IV were from Merck Biosciences. Apyrase, bovine serum albumin (BSA), human and bovine fibrinogen (fraction 1, type III), and non-radioactive protein tyrosine kinase assay kit were all from Sigma. Cangrelor (AR-C69931MX) was kindly provided by The Medicine Company. Ancrod came from NIBSC; fluorescein isothiocyanate (FITC)-labeled annexin A5 from PharmaTarget, and FITC-labeled monoclonal antibody (mAb) against platelet-bound human fibrinogen from WAK Chemie Medical. Rabbit anti-phospho-Syk (Tyr^525/526^) mAb and HRP-linked anti-rabbit IgG were from Cell Signaling Technology, mouse anti-Syk mAb was from Santa Cruz Biotechnology, rabbit anti-α-tubulin Ab from Abcam, and HRP-linked anti-mouse IgG from GE Healthcare. Pefabloc (Gly-Pro-Arg-Pro-amide, GPRP) was obtained from Kordia Life Sciences. Microbeads coated with human anti-CD31 mAb and MS columns were a kind gift from Miltenyi Biotec. Procoagulant phospholipid vesicles (PS:phosphatidyl choline:phosphatidyl ethanolamine 1:3:1; mol/mol) were prepared as described [27]. Convulxin was purified to homogeneity from the venom of *Crotalus durissus terrificus* [28]. Other materials including fibrinogen were from sources indicated before [27].

### Platelet and plasma preparation

Blood was taken from healthy volunteers and from two patients with Glanzmann’s thrombasthenia, with established deficiencies in integrin α_IIb_β_3_ [29], after informed consent and in accordance with the Declaration of Helsinki. Approval was received from the local medical ethical committee. Blood was collected into 1/10 volume of 129 mM trisodium citrate. PRP was obtained by centrifuging at 240*g* for 15 min and platelet-free plasma (PFP) by centrifuging twice at 2,630*g* for 10 min. Platelet count in PRP was determined with a thrombocounter (Coulter Electronics) and normalized with autologous PFP. Citrate-anticoagulated PFP was partly defibrinated by a 10-min treatment with low ancrod protease (1.3 U/mL). After centrifuging the fibrin clots that were formed, non-turbid plasma was isolated. The remaining fibrinogen content was determined at ~0.5 mg/mL according to the conventional Claus method based on turbidimetric measurements [30]. When supplemented with platelets, the ancrod-treated plasma showed normal collagen-induced platelet aggregation responses.

For the preparation of washed platelets, blood was collected into 1/6 volume of acid-citrate glucose solution (ACD, 80 mM trisodium citrate, 52 mM citric acid and 180 mM glucose). Platelets were obtained by centrifugation, washed in the presence of apyrase (0.1 U/mL ADPase), and resuspended in Hepes buffer pH 7.45 (10 mM Hepes, 136 mM NaCl, 2.7 mM KCl, 2 mM MgCl_2_, 0.1 % glucose and 0.1 % BSA) at a count of 1.0 × 10^8^/mL [27].

For experiments with reconstituted PRP, partly defibrinated plasma was supplemented with washed platelets (1.0 × 10^8^/mL). Apyrase was not added, because of the presence of autologous exonucleotidase activity in plasma.

### Flow cytometry

Washed, unstirred platelets in Hepes buffer were activated with PAR1 agonist SFLLRN (15 μM) or thrombin (10 nM) in combination with convulxin (50 ng/mL). Alternatively, the washed platelets were resuspended in ancrod-treated citrate plasma at 1.0 × 10^8^/mL. The reconstituted PRP was activated with tissue factor (2 pM) and CaCl_2_ (16.6 mM) at 37 °C. After 15 min of activation, PS exposure and integrin activation were determined with FITC-labeled annexin A5 or FITC-labeled mAb against platelet-bound human fibrinogen, respectively, using flow cytometry [13]. For cytosolic Ca^2+^ measurements, platelets were loaded with Fura-Red (22 μM) and pluronic (400 μg/mL) in the presence of apyrase (0.1 U/mL ADPase). After a washing step, the loaded platelets were resuspended in ancrod-treated citrate plasma, which was triggered by tissue factor (2 pM) and CaCl_2_ (16.6 mM) at 37 °C. Increases in cytosolic Ca^2+^, apparent as decreases in fluorescence, were recorded in time by flow cytometry [26].

### Thrombin generation

Thrombin generation was determined in normalized PRP (1.5 × 10^8^ platelets/mL) or, as a control, in PFP supplemented with phospholipid vesicles (10 μM). The normalized PRP from control subjects or a Glanzmann patient was activated with tissue factor/CaCl_2_, and fluorescence accumulation was measured according to the thrombogram method under non-stirred conditions in a Fluoroskan Ascent well-plate reader at 37 °C [12]. Nanomolar thrombin concentrations were obtained by comparison with a human thrombin standard using Thrombinoscope software.

### Spectrofluorometry

Platelets were loaded with Fura-2 when rises in cytosolic Ca^2+^ concentration were determined in the absence of plasma [31]. Fura-2-loaded platelets were activated in the presence of 2 mM CaCl_2_ at slow stirring (100 rpm, 37 °C); inhibitors were given before (10 min) or after agonist addition, as indicated. Nanomolar changes in Ca^2+^ level were obtained by calibration procedures, described in detail elsewhere [32].

### Fluorescence microscopy

Glass coverslips were coated with 25 μL of fibrinogen solution (1 mg/mL), rinsed twice with saline, and incubated with washed (Fura-2-loaded) platelets in Hepes buffer pH 7.45 (1.0 × 10^8^/mL) [32]. Where indicated, the coverslips were coated with a low fibrinogen solution (10 μg/mL). After 30 min, non-adherent platelets were removed, and the adhered spreading platelets were stimulated with thrombin (10 nM) in the presence of 2 mM CaCl_2_. Microscopic phase-contrast and fluorescence images of PS exposure (FITC-labeled annexin A5) were taken using a dual camera imaging system, controlled by Visitech software [33]. Fluorescence ratio images of Fura-2 fluorescence were taken to obtain rises in [Ca^2+^]_i_. For calibration, fluorescence values were obtained from Ca^2+^-saturated and Ca^2+^-free lysed platelets containing the fluorescent probe, using the microscopic and camera settings as described [34].

### Platelet isolation from coagulating plasma

Washed platelets (5 × 10^8^ platelets/mL) were reconstituted in ancrod-defibrinated plasma in the presence of GPRP (1 mg/mL) and cangrelor (20 μM). Samples of reconstituted PRP were preincubated with vehicle, Syk inhibitor II (5 μM), and tirofiban (10 μg/mL), as indicated, and activated with tissue factor (2 pM) and CaCl_2_ (16.6 mM). Initial attempts were made to isolate platelets from the activated PRP by centrifugation or gel filtration, but these were unsuccessful. Hence, a novel method was developed, in which platelets were captured from activated PRP by addition of anti-CD31 mAb-coated magnetic microbeads. After 15 min of activation, these platelets were isolated by passage of the PRP through a separation column, and an immediate rinse to remove all plasma proteins. Isolated platelets in the separation column were immediately lysed by flowing with lysis buffer (600 mM NaCl, 40 mM Tris, 4 mM EGTA, 4 mM EDTA, 4 % nonidet-P40, 10 mM Na_3_VO_4_, 4 mM PMSF, 20 μg/mL leupeptin, 20 μg/mL aprotinin, 5 μg/mL pepstatin A). Lysates were frozen at −80 °C until use.

### Protein separation and western blotting

Platelet lysates were separated by polyacrylamide gel electrophoresis and subjected to standard western blotting. Blots were stained for phosphorylated Syk with anti-Syk Tyr^525/526^ mAb (1:1,000) and secondary HRP-conjugated secondary Ab (1:500). Total Syk was determined by reprobing with anti-Syk mAb (1:1,000) and HRP-conjugated secondary Ab (1:1,000). To control for total platelet proteins, parallel blots were probed for α-tubulin (1:1,000). Antibody staining was quantified by densitometric analysis [35].

### Statistics

Data are given as mean ± SEM. Significance of differences was determined with the Mann–Whitney *U* test or the independent samples *t* test, as appropriate, using the statistical package for social sciences (SPSS 15.0).

## Results

### Roles of integrin α_IIb_β_3_ in tissue factor-stimulated thrombin generation and platelet activation

**Fig. 1:**
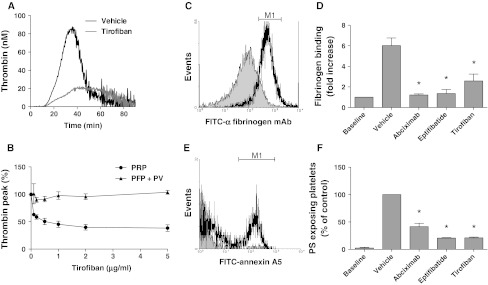
Blocking of integrin α_IIb_β_3_ suppresses PS exposure and thrombin generation in tissue factor-stimulated PRP. **a**, **b** *PRP* (1.0 × 10^8^ platelets/mL) or PFP supplemented with phospholipid vesicles (*PV*, 10 μM) was preincubated with vehicle or tirofiban (0.1–5 μg/mL) for 20 min. Thrombin generation was stimulated with tissue factor (1 pM) and CaCl_2_. **a** Representative thrombin generation curves with PRP, **b** dose-dependent inhibition of thrombin generation only in the presence of platelets. Mean ± SEM (*n* = 3–7). **c**–**f** Ancrod-treated PRP was preincubated with vehicle, abciximab (10 μg/mL), eptifibatide (10 μg/mL) or tirofiban (1 μg/mL), and thrombin generation was stimulated as above. After 15 min, platelet activation was evaluated by flow cytometry. **c**, **d** Platelet fibrin(ogen) binding measured with FITC-labeled anti-fibrinogen mAb. Data are fold increase in fluorescence relative to baseline (prior to activation). **e**, **f** Platelet PS exposure measured with FITC-annexin A5. Data are fractions of PS-exposing platelets (compared to vehicle control). *M1* indicates platelet populations with increased fluorescence. Mean ± SEM (*n* = 3–6); *p < 0.05 versus vehicle

Early reports suggest that various integrin α_IIb_β_3_ antagonists differently affect thrombin generation in platelet-rich plasma (PRP) [19, 23]. To verify this, we prepared human PRP and determined the effects on tissue factor-induced thrombin generation of three α_IIb_β_3_ blockers, all in clinical use: the human/mouse chimeric monoclonal antibody fragment abciximab (blocking integrins α_IIb_β_3_ and α_v_β_3_), the peptide mimetic eptifibatide, and the non-peptide sulfonamido compound, tirofiban. When added to recalcified PRP, tirofiban dose-dependently suppressed and delayed thrombin generation induced by tissue factor (Fig. 1a, b). A similar reduction in thrombin generation was seen with the other α_IIb_β_3_ blockers (Online Resource Fig. 1a, b). Tirofiban, abciximab and eptifibatide reduced the thrombin peak with 60 % at IC_50_ values of 0.1, 2 and 1 μg/mL, respectively. In spite of this reduction, a thrombin peak of 20 nM was still reached, which is sufficiently high for maximal thrombin-induced platelet activation. Control experiments indicated that the integrin blockers were similarly effective in suppressing platelet aggregation of PPACK-anticoagulated PRP, stimulated with the PAR1 agonist SFLLRN (not shown). In contrast, none of the α_IIb_β_3_ blockers affected thrombin generation in plasma containing phospholipids instead of platelets (Fig. 1b and Online Resource Fig. 1b), thus demonstrating that the blocker effects required the presence of platelets.

In order to study tissue factor-induced platelet activation in plasma, formation of disturbing fibrin clots needed to be prevented. Therefore, human plasma was partly defibrinated with a low concentration (1.3 U/mL) of the snake venom ancrod, which induces fibrin clotting without thrombin generation [36]. After removal of the ancrod clots by centrifugation, the remaining plasma contained a residual concentration of ~0.5 mg/mL fibrinogen, which does not form large clots. In this plasma reconstituted with platelets, α_IIb_β_3_ blockers suppressed tissue factor-induced thrombin generation with 40–50 % (data not shown). Flow cytometry demonstrated that, after tissue factor stimulation, fibrin(ogen) binding to platelets was antagonized by any of the three α_IIb_β_3_ blockers (Fig. 1c, d). Furthermore, tissue factor induced PS exposure in 35 % of the platelets, as determined by staining with FITC-annexin A5 (Fig. 1e). In the absence of tissue factor, fibrin(ogen) binding and PS exposure were quite low. Strikingly, blockage of α_IIb_β_3_ greatly reduced the tissue factor-induced PS exposure with 60 % (abciximab) or 80 % (eptifibatide, tirofiban) at maximally effective concentrations (Fig. 1f). Flow cytometry furthermore indicated that the integrin blockers suppressed formation of PS-exposing microparticles by >50 %, as reported before [17]. Similar experiments were performed in the presence of apyrase (0.1 U/mL), in which case again abciximab, eptifibatide, and tirofiban reduced the number of PS-exposing platelets by 75–85 %.

**Fig. 2:**
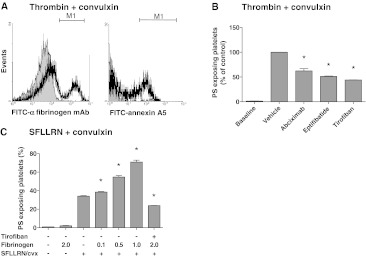
Blocking of integrin α_IIb_β_3_ suppresses PS exposure of convulxin/thrombin-stimulated platelets. Washed platelets containing apyrase were activated with thrombin (10 nM) or SFLLRN (15 μM) plus convulxin (50 ng/mL), as indicated, in the presence of CaCl_2_ (2 mM) for 15 min. Pretreatment with abciximab (10 μg/mL), eptifibatide (10 μg/mL) or tirofiban (1 μg/mL). **a** Histograms of fibrin(ogen) binding (FITC-anti-fibrinogen mAb) and PS exposure (FITC-annexin A5). **b** Quantitative effect of α_IIb_β_3_ blockage on platelet PS exposure. **c** Effect of added human fibrinogen (0.1–2.0 mg/mL) on PS exposure. Mean ± SEM (*n* = 3); *p < 0.05 versus vehicle

A frequently used way of provoking PS exposure is by stimulating washed platelets with thrombin in combination with collagen receptor agonist, convulxin [37]. Considering that these platelets secrete fibrinogen which binds to α_IIb_β_3_ in an autocrine way, we investigated whether in this condition α_IIb_β_3_ blocking may also influence PS exposure. Flow cytometry indicated that the co-stimulation of isolated platelets with thrombin (10 nM) and convulxin (50 ng/mL) resulted in large fractions of platelets binding fibrin(ogen) and exposing PS (Fig. 2a). Dose–response experiments indicated that this concentration of thrombin (10 nM) was maximally effective (not shown), similarly as described before [38]. All integrin blockers caused a substantial decrease in PS exposure of 35 % (abciximab) or 50 % (eptifibatide, tirofiban) of the control condition (Fig. 2b). This suggested a supportive role of α_IIb_β_3_ in thrombin and collagen receptor-induced PS exposure via interaction with (secreted) fibrinogen.

To further study this under conditions where fibrin clot formation was prevented, the platelets were activated with convulxin plus PAR1 agonist SFLLRN. Addition of exogenous human fibrinogen resulted in a dose-dependent stimulatory effect on PS exposure, increasing the fraction of PS-exposing platelets from 35 up to 70% (Fig. 2c). In contrast, addition of fibrinogen alone, without other agonists, did not stimulate PS exposure. Pretreatment with tirofiban (Fig. 2c) or other integrin blockers (not shown) completely reversed the stimulating effect of fibrinogen. Comparable results were obtained with bovine and human fibrinogen (not shown). Together, these results point to a role of integrin α_IIb_β_3_, likely via interaction with fibrin(ogen) on platelet PS exposure both in tissue factor-stimulated PRP (resulting in increased thrombin generation), and in washed platelets stimulated with thrombin and collagen receptor agonists.

### Signaling role of integrin α_IIb_β_3_ in Ca^2+^ and procoagulant platelet responses

**Fig. 3:**
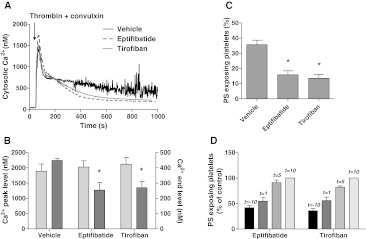
Blocking of α_IIb_β_3_ suppresses long-term platelet Ca^2+^ responses and PS exposure. Fura-2-loaded platelets containing apyrase were preincubated with vehicle (control), eptifibatide (10 μg/mL) or tirofiban (1 μg/mL). Cells were then activated with thrombin (10 nM) plus convulxin (50 ng/mL) and 1 mM CaCl_2_. **a** Representative platelet Ca^2+^ responses. **b** Effect of integrin blockers on Ca^2+^ peak levels (*light gray*) and 15-min end levels (*dark gray*). **c** Effect of blockers on fractions of PS-exposing platelets after 15 min, analyzed by flow cytometry. **d** Effect of addition of eptifibatide or tirofiban at different time points before (*t* = −10 min) or after (*t* = 1–10 min) activation. Fractions of PS-exposing platelets after 15 min. Data are relative to control condition without integrin blocker. Mean ± SEM (*n* = 3–5); *p < 0.05 versus vehicle

Others have suggested that inhibitory effects of α_IIb_β_3_ blockers on platelet PS exposure may occur independently of modulating Ca^2+^ responses [22, 39]. Also, in stored platelets, PS exposure can occur independently of elevated Ca^2+^ [40]. We therefore re-examined a role of α_IIb_β_3_ in Ca^2+^-signaling by stimulating washed suspensions of Fura-2-loaded platelets with thrombin and convulxin. While measuring rises in Ca^2+^, samples were taken for flow cytometric determination of PS exposure. The agonists caused a potent increase in Ca^2+^ peak, which was followed by a sustained elevated level (450 nM), persisting during 15 min (Fig. 3a). Pretreatment with eptifibatide or tirofiban did not influence the initial Ca^2+^ peak, but it markedly reduced the sustained high Ca^2+^ level to 56 ± 10 or 60 ± 8 % of the control value, respectively (Fig. 3a, b). The reduction in sustained Ca^2+^ response was accompanied by a proportional decrease in PS exposure from 36 ± 3 to 16 ± 3 or 14 ± 3 %, respectively (Fig. 3c). Reasoning that persistent integrin signaling may prolong these Ca^2+^ responses and then contribute to PS exposure, we added the integrin blockers at various time points after thrombin/convulxin. Addition of eptifibatide or tirofiban at 1 min after activation still caused a substantial reduction in the fraction of PS-exposing platelets, whereas addition after 5–10 min resulted in progressively less inhibition (Fig. 3d).

**Fig. 4:**
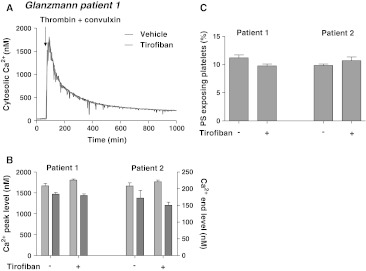
Impaired long-term Ca^2+^ responses and PS exposure in activated Glanzmann platelets. Suspensions of Fura-2-loaded platelets containing apyrase from two Glanzmann patients (1, 2) were pretreated with vehicle or tirofiban (1 μg/mL). Platelets were then activated with thrombin (10 nM) plus convulxin (50 ng/mL) in the presence of 1 mM CaCl_2_. **a** Representative traces of Ca^2+^ responses. **b** Averaged Ca^2+^ peak levels (*light gray*) and 15-min end levels (*dark gray*). **c** Fractions of PS-exposing platelets after 15 min, showing no effect of tirofiban. Mean ± SEM (*n* = 3 experiments)

To further confirm the contribution of integrin signaling in PS exposure, platelets were obtained from two Glanzmann patients with complete deficiency in α_IIb_β_3_ expression. Loaded with Fura-2, the platelets showed high peak rises in Ca^2+^ in response to thrombin/convulxin, but at later time points Ca^2+^ levels declined to ~150 nM (Fig. 4a, b). This corresponded to a low amount of 10–12 % PS-exposing platelets (Fig. 4c). Addition of tirofiban altered neither the late Ca^2+^ response nor the low exposure of PS. Collectively, these data point to a role of α_IIb_β_3_-mediated signaling in long-term Ca^2+^ rises induced by thrombin and collagen receptor agonists and thereby in development of platelet procoagulant activity.

### Signaling role of integrin α_IIb_β_3_ in Ca^2+^ and procoagulant responses of platelets spreading on fibrinogen

**Fig. 5:**
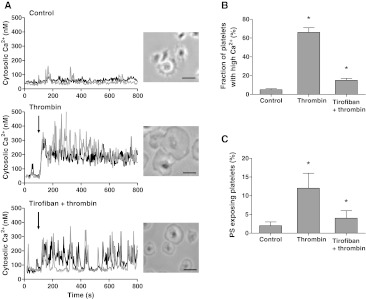
Blocking of integrin α_IIb_β_3_ reduces Ca^2+^ responses and PS exposure of spreading platelets. Fura-2-loaded platelets were adhered to coated fibrinogen in the absence (control) or presence of tirofiban (1 μg/mL) for 30 min. Rises in Ca^2+^ in single, adhered platelets in response to thrombin (10 nM) measured by fluorescence ratio imaging. **a** Traces of Ca^2+^ rises of two representative platelets; also brightfield contrast images after 10 min (*bars* 10 μm). **b** Fractions of platelets with elevated Ca^2+^ (>1.2-fold signal) after 10 min. **c** Fractions of platelets binding FITC-annexin A5 after 10 min. Mean ± SEM (*n* = 4–6); *p < 0.05 versus control

Outside-in signaling by integrin α_IIb_β_3_ mediates the spreading of platelets on fibrinogen surfaces [4]. In Fura-2-loaded platelets, we found that spreading was accompanied by irregular, low-amplitude Ca^2+^ spikes (Fig. 5a). In most of these platelets, thrombin induced a persistently high Ca^2+^ level and stimulated the spreading process (Fig. 5a, b). To investigate a role of integrin signaling, the platelets were preincubated with tirofiban prior to thrombin stimulation. This retarded the spreading process, and suppressed the Ca^2+^ response, in a way that the persistent Ca^2+^ rise changed into a pattern of repetitive Ca^2+^ spiking. Furthermore, tirofiban reduced the fractions of PS-exposing platelets with thrombin from ~15 to only 2.5 % (Fig. 5c). The inhibiting effects of tirofiban were preserved on a surface coated with a low fibrinogen solution of 10 μg/mL (not shown). Thrombin stimulation of adhered, non-spread platelets (5 min fibrinogen adhesion) similarly resulted in a low fraction of PS-exposing platelets of 2.5 ± 0.4 %. Control experiments were carried out with DM-BAPTA-loaded platelets, where basal levels of Ca^2+^ amounted ~20 nM, and thrombin addition resulted in neither Ca^2+^ rises nor PS exposure (data not shown).

To further verify a role of α_IIb_β_3_ in thrombin-induced responses of fibrinogen-adhered platelets, similar experiments were performed with the platelets from two Glanzmann patients. While these platelets hardly spread on fibrinogen, they also remained low in PS exposure with only ~2 % annexin A5 binding after thrombin stimulation (Online Resource Fig. 2). These data thus suggest that integrin outside-in signaling during platelet spreading stimulates thrombin-induced procoagulant activity.

### Contribution of integrin α_IIb_β_3_ to platelet procoagulant response via Syk kinase activation

**Fig. 6:**
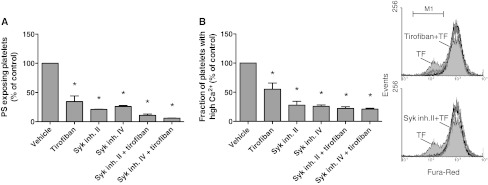
Contribution of Syk kinase to platelet activation in tissue factor-stimulated plasma. Fura-Red loaded platelets in ancrod-treated plasma (1.0 × 10^8^/mL) were preincubated with vehicle, Syk inhibitor II or IV (10 μM), and/or tirofiban (1 μg/mL), as described for Fig. 1. Cangrelor (AR–C, 10 μM) was present to eliminate P2Y_12_-dependent signaling events. PRP was then stimulated with 1 pM tissue factor and CaCl_2_ for 15 min. **a** Fractions of PS-exposing platelets determined by FITC-annexin A5 binding. **b** Fractions of platelets with high Ca^2+^ as determined by flow cytometry (*M1*). Flow cytometric histograms of Fura-Red fluorescence. Note decreased Fura-Red fluorescence points to high Ca^2+^ (*M1*). Histograms of unstimulated platelets (*black*), and tissue factor-stimulated platelets with vehicle (*light gray*), tirofiban or Syk inhibitor II (*dark gray*). Mean ± SEM (*n* = 4); *p < 0.05 versus vehicle

Integrin α_IIb_β_3_-mediated outside-in signaling triggers inactivation of RhoA and activation of the protein tyrosine kinase Syk, resulting in phospholipase Cγ2 activation [6, 41]. Employing several approaches, we investigated a role of Syk in α_IIb_β_3_-dependent Ca^2+^ rises and procoagulant activity. First, we used the pharmacologic blockers, Syk inhibitor II and IV, which abolished collagen-induced aggregation of platelets in plasma at maximally effective concentrations of 10 μM. In tissue factor-stimulated PRP, both inhibitors suppressed platelet PS exposure at a similar degree as tirofiban (Fig. 6a). Next, platelets were loaded with the Ca^2+^ probe Fura-Red, which allows the monitoring of Ca^2+^ rises in the presence of plasma by flow cytometry [42]. Stimulation with tissue factor resulted in a prolonged rise in Ca^2+^ in the majority of platelets in plasma (observed as a decrease in Fura-Red fluorescence). This Ca^2+^ rise was reduced in the presence of tirofiban and even more so with Syk inhibitor II or IV (Fig. 6b).

**Fig. 7:**
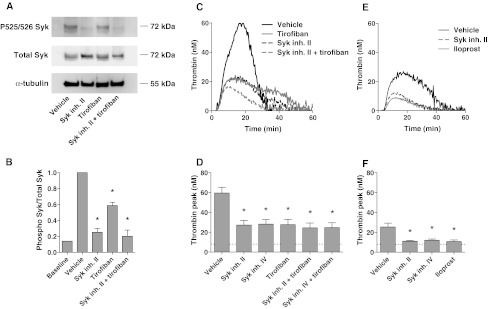
Contribution of integrin α_IIb_β_3_ signaling and Syk kinase to thrombin generation in tissue factor-stimulated plasma. Reconstituted PRP was preincubated with vehicle, Syk inhibitor II (10 μM), iloprost (1 μM) and/or tirofiban (1 μg/mL), as indicated, and then stimulated with tissue factor (1 pM) and CaCl_2_. Cangrelor (AR–C, 10 μM) was present to eliminate P2Y_12_-dependent signaling events. Control condition (*baseline*) was without tissue factor. **a**, **b** Effects of tirofiban and Syk inhibitor on Tyr^525/526^ phosphorylation of Syk. Platelets were isolated from activated plasma (15 min) using anti-CD31-coupled magnetic beads and examined for protein phosphorylation. Representative western blots after probing for phospho-Syk (Tyr^525/526^), and reprobing for total Syk; parallel blots were stained for α-tubulin. Shown is the ratio of phospho-Syk/total Syk assessed by densitometric analysis (*n* = 6). **c**–**f** Effects of tirofiban and Syk inhibitor on tissue factor-induced thrombin generation in PRP. Blood was collected on citrate (*n* = 8) (**c**, **d**); or on citrate plus tirofiban (1 μg/mL) (*n* = 3) (**e**, **f**). Representative thrombin generation curves and thrombin peak heights. *Dotted lines* in *bar graphs* indicate residual thrombin formed in plasma not containing platelets. Mean ± SEM; *p < 0.05 versus vehicle

Third, we directly examined the activation of Syk in tissue factor-stimulated PRP. It appeared not to be possible to collect these platelets from plasma by centrifugation or gel filtration. Hence, we developed a method to isolate platelets after tissue factor stimulation by using magnetic beads coupled to anti-CD31 mAb. Lysates of the isolated platelets were subjected to gel electrophoresis and western blotting, and probed for phosphorylation of Syk at Tyr^525/526^, which is an essential step in Syk activation [43]. While no Syk phosphorylation was detected in the absence of tissue factor, platelet stimulation with tissue factor stimulation markedly increased the phosphorylation, which event was completely prevented by Syk inhibitor II (Fig. 7a, b). Pretreatment with tirofiban substantially but not completely antagonized Tyr^525/526^ phosphorylation, suggesting a prominent role of α_IIb_β_3_-dependent signaling to Syk in the tissue factor-activated PRP.

Fourth, we directly investigated the effects of Syk inhibitors II and IV on tissue factor-induced thrombin generation. Using citrate-anticoagulated PRP, it appeared that both compounds were similarly effective as tirofiban in the suppression of thrombin generation, while there was no additional effect of a combination with tirofiban (Fig. 7c, d). As platelet-dependent thrombin generation was still incompletely blocked, we considered the possibility that the platelets exhibited residual α_IIb_β_3_-dependent signaling during blood collection and PRP preparation. To investigate this, blood was collected on citrate anticoagulant plus tirofiban. Indeed, with tirofiban initially present, tissue factor-induced thrombin generation was reduced, while either Syk inhibitor fully reduced the thrombin peak to the level obtained with the strong platelet inhibitor, iloprost (Fig. 7e, f). In fact, this residual, low thrombin generation was also present in plasma devoid of platelets, and could be ascribed to the presence of microparticles. Similar results were obtained with blood collected on Syk inhibitor II (not shown).

Finally, thrombin generation experiments were performed with PRP from a Glanzmann patient. In the patient PRP, tissue factor stimulation evoked limited thrombin generation, which, however, was not influenced by the presence of tirofiban (Online Resource Fig. 3). Similar to control PRP with tirofiban, addition of Syk inhibitor caused an additional decrease in thrombin generation. Taken together, these various sets of data show a substantial role of α_IIb_β_3_ and Syk kinase in tissue factor-induced PS exposure and thrombin generation. Furthermore, they point to the existence of a pathway of thrombin generation that is dependent on Syk, but not on integrin activation.

## Discussion

This paper reveals a new role of integrin α_IIb_β_3_-dependent signaling via Syk kinase in tissue factor-induced platelet procoagulant activity and thrombin generation in plasma. The results point to a pathway where initial traces of thrombin triggered by tissue factor activate platelets to expose procoagulant PS, resulting in a cycle of thrombin generation and platelet activation that is greatly enforced and prolonged by integrin-dependent signaling to Syk activation and Ca^2+^ rises. The data furthermore identify a Syk-dependent, but integrin-independent pathway of thrombin generation.

In tissue factor-stimulated PRP, we found that the blockage of α_IIb_β_3_ with different antagonists suppresses platelet Ca^2+^ responses, PS exposure and thrombin generation in a dose-dependent way. However, in washed platelets stimulated via thrombin and collagen receptors, integrin blockage also suppressed long-term Ca^2+^ rises along with PS exposure, while added fibrinogen enhanced these responses. Furthermore, during platelet spreading on fibrinogen, a process known to rely on α_IIb_β_3_ outside-in signaling, blocking of the integrin resulted in Ca^2+^ signaling and PS exposure in response to maximally effective concentrations of thrombin. Confirmative evidence for a signaling role of α_IIb_β_3_ came from the observation that long-term Ca^2+^ responses and PS exposure were reduced in platelets from two patients with Glanzmann’s thrombasthenia, lacking α_IIb_β_3_. Together, these data indicate that, in platelets stimulated with Ca^2+^ mobilizing agonists, α_IIb_β_3_ outside-in signaling prolongs the Ca^2+^ signal, increases procoagulant activity, and hence supports tissue factor-stimulated thrombin generation on the platelet surface.

Anticoagulant effects of platelet α_IIb_β_3_ antagonists have been reported by several authors [12, 16, 17], but the mechanism was not disclosed. Several groups reported that integrin blockers were unable to change platelet Ca^2+^ responses to collagen and/or thrombin [22, 23, 39]. However, the measurements mostly concerned initial Ca^2+^ rises, while in our hands only late Ca^2+^ signals appear to be affected. Interestingly, one study does describe long-term inhibition of collagen/thrombin-induced Ca^2+^ responses with abciximab but not with other integrin blockers specifically under conditions of stirring [23]. This contrasts with the present findings where appropriate concentrations of different integrin blockers all had similar effects. Another published finding that α_IIb_β_3_ blockage reduces shear-dependent Ca^2+^ responses and microparticle release [19, 44] can be explained by increased fibrinogen secretion of platelets subjected to a high shear rate.

The (patho)physiological relevance of this work comes from recent data that β_3_-mutated mice with a deficiency in platelet outside-in signaling and tyrosine phosphorylation are protected from arterial thrombus formation after carotid artery injury with FeCl_3_ [45], i.e. a mouse thrombosis model where thrombus formation depends on tissue factor activity and on procoagulant, PS-exposing platelets [13, 46–48].

Platelets from mice deficient in phospholipase Cγ2 have shown reduced Ca^2+^ signals and spreading on immobilized fibrinogen [44, 49]. Based on these and other data a scheme has been proposed of α_IIb_β_3_-induced signaling via Src and Syk kinases to activation of phospholipase Cγ2 [6]. This signaling scheme was confirmed by recent proteomic analyses demonstrating the presence of many tyrosine phosphorylated proteins in human platelets spread on fibrinogen, among which multiple tyrosine kinases [5]. The present finding points to a particular role of Syk phosphorylation and subsequent phospholipase Cγ2 activation in α_IIb_β_3_-dependent procoagulant activity and thrombin generation upon triggering with tissue factor. Evidence for this came from the reduction in Syk tyrosine phosphorylation by integrin blockage and by two Syk inhibitors. These experiments were carried out under conditions eliminating a contribution of P2Y_12_ signaling [24, 50].

Interestingly, in addition to a novel α_IIb_β_3_-dependent role of Syk kinase in platelet procoagulant activity, our data also point to an α_IIb_β_3_-independent role of Syk in this process. We have not yet unraveled the mechanism of α_IIb_β_3_-independent Syk activation, but according to the literature this activation pathway can include FcγRIIa [51], glycoprotein Ib–IX–V complex [52, 53], or CLEC2 [54], all of which have been shown to activate Syk. Altogether, our results point to a signaling scheme where fibrin(ogen)-induced integrin activation supports a Syk/phospholipase Cγ2 pathway, resulting in prolonged Ca^2+^ and PS exposure and thrombin generation in plasma.

## Electronic supplementary material

Below is the link to the electronic supplementary material.

Supplementary material 1 (PDF 267 kb)
