# Supplementary material for: Key role of integrin αIIbβ3 signaling to Syk kinase in tissue factor-induced thrombin generation
Source: Cell Mol Life Sci. 2012 Jun 6;69(20):3481–92. doi: 10.1007/s00018-012-1033-2 (PMC3445795; doi:10.1007/s00018-012-1033-2)
Supplement: Supplementary file 1 — Supplementary material 1 (PDF 267 kb) [file 18_2012_1033_MOESM1_ESM.pdf]

Electronic Supplementary Material

Cellular and Molecular Life Sciences

Key role of integrin $\alpha_{IIb}\beta_3$ signaling to Syk kinase in tissue factor-induced thrombin generation

Paola E.J. van der Meijden[1], Marion A.H. Feijge[1*], Frauke Swieringa[1*], Karen Gilio[1], Reyhan Nergiz-Unal[1], Karly Hamulyák[2] and Johan W.M. Heemskerk[1**]

[1]Department of Biochemistry, Cardiovascular Research Institute Maastricht (CARIM); [2]Haemostasis Laboratory, Department of Internal Medicine, Maastricht University Medical Centre, Maastricht, The Netherlands.

*M.A.H.F. and F.S. contributed equally

**Corresponding author: Johan W. M. Heemskerk PhD, Dep. Biochemistry (CARIM), Maastricht University, PO Box 616, 6200 MD Maastricht, The Netherlands.

Tel: +31-43-3881671, fax: +31-43-3884159; e-mail: jwm.heemskerk@maastrichtuniversity.nl

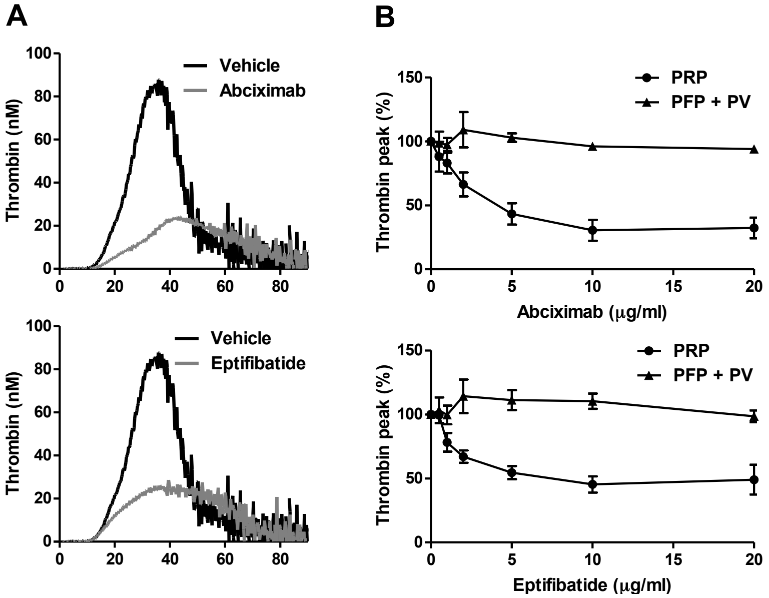

Fig. 1 Blocking of $\alpha_{IIb}\beta_3$ suppresses tissue factor-triggered thrombin generation in the presence of platelets. Human PRP (1.0×10^8 platelets/mL) or PFP supplemented with phospholipid vesicles (PV, 10 μM) was preincubated with vehicle, abciximab (0.5-20 μg/mL) or eptifibatide (0.5-20 μg/mL) for 20 minutes. Thrombin generation was stimulated with tissue factor (1 pM) and $CaCl_2$. **a** Representative thrombin generation curves with PRP, showing comparable effects of all integrin blockers. **b** Dose-dependent inhibition of thrombin generation only in the presence of platelets. Means ± SEM (*n*=3-9)

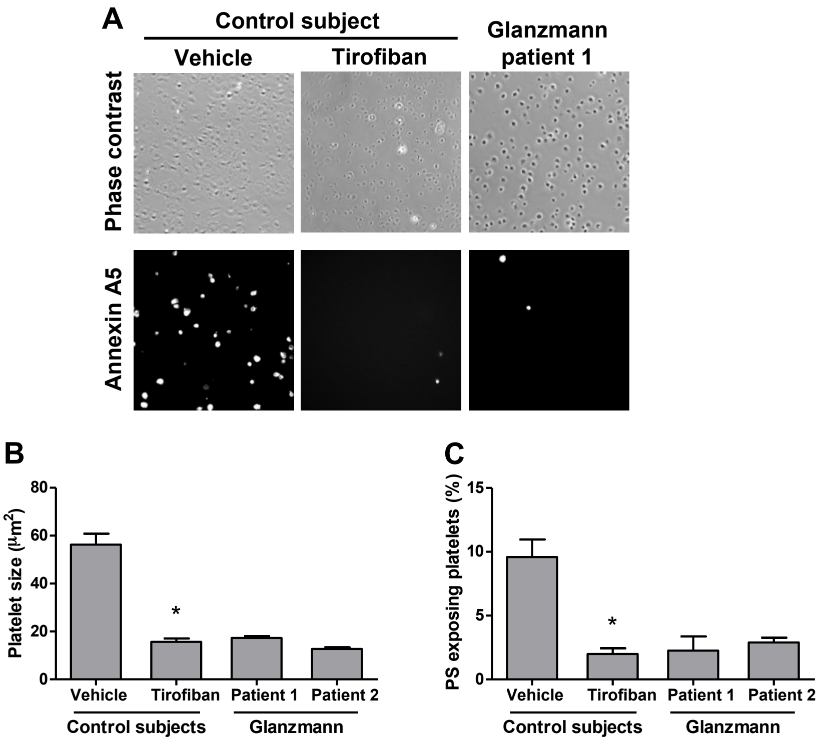

Fig. 2 Impaired spreading and thrombin activation of Glanzmann platelets. Platelets from healthy control subjects and Glanzmann patients were adhered to fibrinogen in the absence (control) or presence of tirofiban (1 μg/mL) for 30 minutes, and then stimulated with thrombin (10 nM) for another 10 minutes. **a** Representative phase contrast (120×120 μm) and FITC-annexin A5 fluorescence (150×150 μm) images after thrombin activation. **b** Averaged platelet size; **c** fractions of PS-exposing platelets after activation. Means ± SEM (*n*=3-6); **p*<0.05 *vs.* vehicle

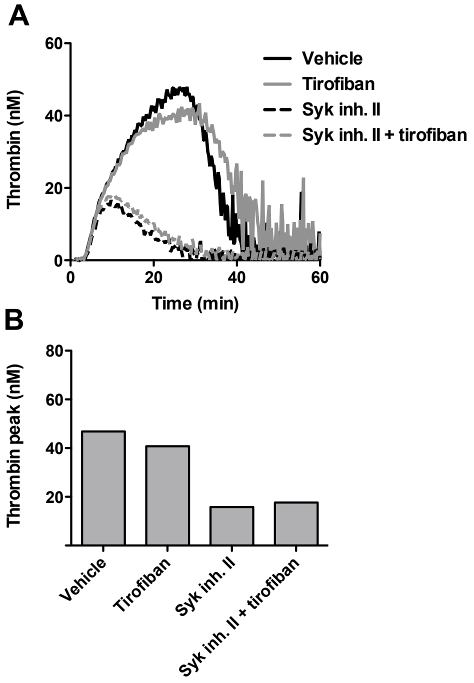

Fig. 3 Syk kinase is responsible for thrombin generation in tissue factor-stimulated PRP of a Glanzmann patient. PRP was incubated with vehicle, Syk inhibitor II (10 μM), iloprost (1 μM) and/or tirofiban (1 μg/mL), and then stimulated with tissue factor (1 pM) and $CaCl_2$. Cangrelor (AR-C, 10 μM) was present to eliminate $P2Y_{12}$-dependent signaling events. **a, b** Effect of inhibitors on tissue factor-induced thrombin generation in PRP from blood samples collected on citrate, and **c, d** on citrate and tirofiban (1 μg/mL). Representative thrombin generation curves and mean thrombin peak heights of triplicate measurements are shown.
